# FSP1 as the convergent hub of autophagy and ubiquitination governing ferroptosis in monocrotaline‐induced pulmonary arterial hypertension

**DOI:** 10.1002/ctm2.70822

**Published:** 2026-11-05

**Authors:** Dandan Chen, Qi Jin, Lei Zhang, Wenzhi Pan, Daxin Zhou, Junbo Ge, Lihua Guan

**Affiliations:** ^1^ Department of Cardiology, Zhongshan Hospital, Shanghai Institute of Cardiovascular Diseases Fudan University Shanghai China; ^2^ National Clinical Research Center for Interventional Medicine Shanghai China

**Keywords:** autophagy, ferroptosis, FSP1, pulmonary arterial hypertension (PAH), ubiquitination

## Abstract

**Introduction:**

The interaction mechanism between autophagy and ferroptosis in pulmonary arterial hypertension (PAH) remains unclear. We identified FSP1 as a core molecular node linking these two critical processes. Mechanistically, the degradation of FSP1 is coordinately regulated by NBR1‐mediated autophagic flux and RNFT1‐driven K63‐linked ubiquitination, collectively exacerbating ferroptosis and PAH progression. Combination therapy involving FSP1 overexpression and the autophagy inhibitor Apelin‐13 synergistically alleviates PAH in rats by stabilizing FSP1 and suppressing ferroptosis. This study not only reveals a dual‐pathway mechanism regulating FSP1 stability and validates a novel therapeutic strategy targeting FSP1 degradation but also provides a novel combined intervention strategy for the clinical treatment of PAH.

**Background and objective:**

The interaction between autophagy and ferroptosis in pulmonary arterial hypertension (PAH) remains poorly understood. This study aims to elucidate the role of ferroptosis suppressor protein 1 (FSP1) as a central molecular node linking these processes and to explore a combined therapeutic strategy targeting FSP1 degradation for PAH intervention.

**Methods:**

Molecular biology techniques were employed to analyse FSP1 degradation pathways, focusing on NBR1‐mediated autophagic flux and RNFT1‐driven K63‐linked ubiquitination. A rat PAH model was established to evaluate the therapeutic efficacy of combining FSP1 overexpression with the autophagy inhibitor Apelin‐13.

**Results:**

FSP1 stability was coordinately regulated by autophagy and ubiquitination: NBR1 promoted autophagic degradation of FSP1, while RNFT1 exacerbated this process via K63‐linked ubiquitination, collectively accelerating ferroptosis and PAH progression. The combination of FSP1 overexpression and Apelin‐13 synergistically stabilized FSP1, suppressed ferroptosis, and alleviated PAH symptoms in rats.

**Conclusion:**

This study reveals a dual‐pathway mechanism governing FSP1 stability through autophagy‐ubiquitination crosstalk, highlighting its critical role in ferroptosis‐driven PAH. The combination therapy targeting FSP1 degradation presents a novel strategic approach for clinical PAH treatment.

## Graphical abstract

Background: The crosstalk between autophagy and ferroptosis in pulmonary arterial hypertension (PAH) remains unclear. We investigated the role of FSP1 as a critical link between these processes.

Methods and Findings: Mechanistically, we identified that FSP1 degradation is dually regulated by NBR1‐mediated autophagic flux and RNFT1‐dependent K63‐linked ubiquitination, collectively promoting ferroptotic cell death and accelerating PAH progression.

Intervention and Outcome: Therapeutic intervention using a combination of FSP1 overexpression and the autophagy inhibitor Apelin‐13 synergistically stabilized FSP1 protein levels, suppressed ferroptosis and effectively ameliorated PAH in rat models.

Conclusion: Our work unveils a novel dual‐pathway mechanism controlling FSP1 stability and provides a promising combined therapeutic strategy targeting FSP1 degradation for the clinical treatment of PAH.

Proposed molecular mechanism of FSP1 degradation in the regulation of ferroptosis and pulmonary arterial hypertension (PAH).

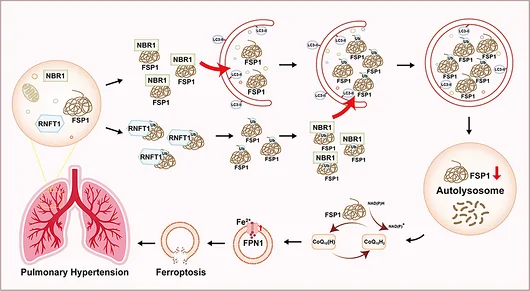

## 1 INTRODUCTION

Pulmonary arterial hypertension (PAH) is a fatal disease characterized by progressive vascular remodelling and increased pulmonary vascular resistance. Its core pathological processes involve abnormal contraction, excessive proliferation, apoptosis resistance and inflammatory responses of pulmonary vascular cells.^1^, ^2^ Although existing vasodilators can alleviate symptoms to some extent, they are unable to reverse the disease progression.^3^, ^4^ Therefore, elucidating novel pathogenic mechanisms of PAH has become an urgent priority in current research.

In recent years, ferroptosis, an iron‐dependent form of regulated cell death driven by excessive lipid peroxide accumulation, has been identified as a key amplifier in the pathogenesis of PAH.^5^ It disrupts vascular integrity, exacerbates inflammatory responses, triggers abnormal proliferation and dysregulates vascular tone, thereby amplifying initial injury signals into irreversible pulmonary vascular remodelling and hypertension.^6^, ^7^, ^8^ Targeted intervention in the ferroptosis pathway holds promise as a potential novel therapeutic strategy for PAH by disrupting this vicious cycle.^9^

The role of autophagy in PAH is complex and context‐dependent.^10^ Moderate autophagy can serve as a protective mechanism by clearing damaged organelles to maintain cellular homeostasis.^11^, ^12^ However, under the chronic stress conditions of PAH, sustained and excessive activation of autophagy promotes disease progression through the degradation of key cellular components.^13^, ^14^ Investigating the interaction between aberrant autophagy activation and ferroptosis provides a novel perspective for understanding the pathological essence of PAH.^7^

Ferroptosis suppressor protein 1 (FSP1) is a key ferroptosis inhibitor independent of the classical glutathione‐GPX4 pathway.^15^ It maintains redox balance via the FSP1/CoQ10/NAD(P)H system, directly suppresses ferroptosis, and is crucial for cellular iron homeostasis.^16^, ^17^ Recent research has comprehensively examined the regulatory framework of FSP1 concerning oxidative stress, lipid metabolism, and diverse pathologies, providing a theoretical basis for designing FSP1‐targeted therapeutics.^18^ Nonetheless, the precise regulatory mechanisms and functional implications of FSP1 in pulmonary arterial hypertension remain obscure.

Selective autophagy and the ubiquitin‐proteasome system are two central pathways regulating specific protein stability.^19^, ^20^ The selective autophagy receptor NBR1 recognizes ubiquitinated substrate proteins via its UBA domain and recruits them to autolysosomes for degradation by binding to LC3 on autophagosomal membranes through its LIR domain.^21^, ^22^, ^23^ RNFT1 is a polytopic transmembrane protein containing a canonical C3HC4‐type RING‐HC finger domain—a signature structural motif of E3 ubiquitin ligases, which strongly implies that RNFT1 harbours intrinsic E3 ubiquitin ligase catalytic activity.^24^ Notably, E3 ubiquitin ligases are well documented to preferentially ubiquitinate substrates destined for recognition and turnover by selective autophagy receptors.^25^Therefore, systematically elucidating how these two degradation pathways regulate specific target proteins will contribute to a more comprehensive understanding of the dynamic protein homeostasis network during PAH development.^26^

This study establishes FSP1 degradation—induced by autophagy activation—as a key mechanism driving PAH through ferroptosis. Loss of FSP1 disrupts the FSP1‐CoQ10‐NAD(P)H axis, leading to iron dyshomeostasis and lipid peroxidation. We further identify NBR1 and RNFT1 as key regulators of FSP1 stability and propose a novel therapeutic strategy combining FSP1 upregulation and autophagy inhibition, providing a foundation for targeting FSP1 degradation in PAH treatment.

## 2 MATERIALS AND METHODOLOGY

In this study, a PAH model was established in Sprague–Dawley (SD) rats, followed by relevant pharmacological interventions. Disease progression was assessed by evaluating haemodynamic and morphological parameters, pulmonary vascular remodelling, inflammatory responses, expression of adhesion molecules and BMPR‐II signalling. Primary rat pulmonary arterial endothelial cells (PAECs) were employed to simulate the PAH pathological microenvironment in vitro. HEK‐293T cells, owing to their high transfection efficiency, were used for mechanistic investigations, including exogenous gene overexpression, co‐immunoprecipitation (Co‐IP) and ubiquitination assays. Autophagic flux was evaluated by measuring LC3‐II and p62 levels under oxidative stress using Western blot or immunofluorescence. Cell proliferation was monitored via CFDA‐SE staining coupled with flow cytometric analysis. Ferroptosis‐related indicators were systematically assessed by quantifying lipid reactive oxygen species (Lip‐ROS) using the C11‐BODIPY 581/591 probe, intracellular Fe^2^^+^ with the FerroOrange probe, malondialdehyde (MDA) content via the thiobarbituric acid (TBA) method, and glutathione (GSH) levels using a commercial kit. For all immunofluorescence, transmission electron microscopy, Matrigel tube formation, H&E and Masson's trichrome staining images shown in main and supplementary figures: representative micrographs were randomly selected from independent biological replicates. These images display typical pathological and cellular phenotypes consistent with the averaged quantitative statistical results of all samples. In vivo experiments contained ≥6 biological replicates per group; in vitro cell assays were repeated at least 3 independent times. All quantitative measurements were normalized, and detailed experimental procedures are provided in the Supporting Information.

## 3 Results

### 3.1 FSP1 is a key gene promoting PAH development by mediating ferroptosis via autophagy activation

To investigate the correlation between autophagy and ferroptosis during the development of PAH, we analysed the GSE117261 dataset. Our results revealed that in PAH samples with active autophagy, ferroptosis was more pronounced, and autophagy levels showed a significant positive correlation with ferroptosis levels (Figure S1A,B). To further identify key molecules involved in PAH pathogenesis through autophagy, we compared differentially expressed genes associated with varying autophagy levels and PAH progression. Our analysis identified ferroptosis suppressor protein FSP1 as a critical molecule linking autophagy and ferroptosis (Figure S1C–E).

To overcome the limitation of relying merely on bulk RNA‐seq data and validate cell‐type‐specific expression alteration of FSP1 in pulmonary arterial endothelial cells (PAECs), we further performed single‐cell transcriptomic analysis based on publicly available dataset GSE210248. UMAP dimensionality reduction clearly distinguished cell subtypes isolated from lung tissues of healthy donors and PAH patients (Figure S1F,G). Cellular fraction annotation confirmed that *AIFM2* was predominantly transcribed in pulmonary endothelial populations, especially endo1 and endo2 subsets (Figure S1H,I). Average expression and proportion statistics illustrated that both transcriptional abundance and positive cell percentage of *AIFM2* were profoundly decreased in endothelial cells derived from PAH lesions (Figure S1J,K). Pseudobulk quantification further validated that overall *AIFM2* expression was significantly lower in PAH patients compared with healthy donors (*p* = 0.04) (Figure S1L), consistent with our bulk transcriptome findings.

In a monocrotaline (MCT)‐induced rat model of PAH,^27^ we evaluated whether Apelin‐13 influences PAH progression by modulating ferroptosis, which exerts its autophagy‐inhibitory effect through activation of the PI3K/Akt/mTOR pathway.^28^ Compared with the Control group, MCT‐PH rats exhibited obvious pulmonary arteriolar medial thickening, excessive collagen deposition and luminal stenosis, which were significantly reversed by Apelin‐13 treatment. Immunohistochemical staining showed increased α‐SMA‐positive smooth muscle proliferation and reduced CD31‐positive endothelial coverage in the MCT‐PH group, while Apelin‐13 effectively restored endothelial integrity and inhibited abnormal vascular remodelling (Figure 1A, Figure S1M). Quantitative analysis revealed a significantly increased pulmonary vascular occlusion rate and elevated right ventricular hypertrophy index (RVH%) in MCT‐PH rats, indicating severe pulmonary vascular obliteration and adaptive right ventricular hypertrophy. Apelin‐13 markedly reduced vascular occlusion and attenuated right ventricular hypertrophic remodelling (Figure 1B). Haemodynamic detection demonstrated that mPAP, PVR, RVSP and RVEDP were significantly increased in the MCT‐PH group, indicative of pulmonary circulation overpressure and right ventricular diastolic dysfunction. Apelin‐13 intervention substantially normalized these core haemodynamic parameters and relieved vascular stenosis‐mediated right ventricular pressure overload (Figure 1F). At the systemic and cardiac functional level, MCT‐PH rats displayed decreased body weight, shortened pulmonary acceleration time and reduced cardiac output (CO). In contrast, Apelin‐13 treatment restored body weight gain, prolonged pulmonary acceleration time and rescued cardiac output, thereby ameliorating chronic pressure overload‐induced right ventricular dysfunction (Figure 1C).

**FIGURE 1:**
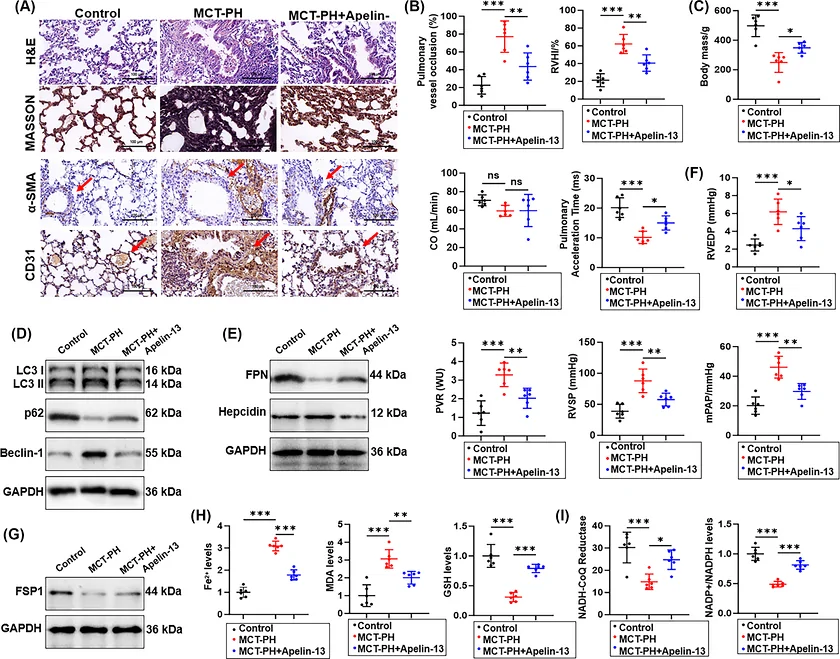
Apelin‐13 ameliorates monocrotaline‐induced pulmonary arterial hypertension, suppresses pathological autophagy, restores FSP1 expression and mitigates endothelial ferroptosis in vivo. (A) Representative H&E and Masson's trichrome staining of lung tissue for pulmonary vascular structural assessment; immunohistochemical staining for α‐SMA (vascular smooth muscle proliferation marker) and CD31 (endothelial integrity marker). Scale bars are indicated on images. (B) Quantitative statistics of pulmonary vessel occlusion rate and right ventricular hypertrophy index (RVH%). (C) Quantitative analysis of body weight, pulmonary acceleration time and cardiac output (CO). (D) Western blot detection of autophagy‐related proteins LC3‐I/LC3‐II, p62 and Beclin‐1 in pulmonary vascular tissues, with GAPDH as internal reference. (E) Immunoblot analysis for iron homeostasis regulators FPN and hepcidin. (F) Haemodynamic quantification of mean pulmonary arterial pressure (mPAP), pulmonary vascular resistance (PVR), right ventricular systolic pressure (RVSP) and right ventricular end‐diastolic pressure (RVEDP). (G) Western blot measurement of FSP1 protein abundance. (H) Biochemical quantification of intracellular ferrous ion concentration, MDA level and GSH content reflecting ferroptotic activity. (I) Detection of NADH‐CoQ reductase activity and NADP^+^/NADPH redox ratio. Data are presented as mean ± SD. Statistical differences were analysed by one‐way ANOVA followed by Tukey's post‐hoc test; *p < .05, **p < .01, ***p < .001; ns, not significant.

Western blot analysis for autophagy and ferroptosis activation revealed elevated LC3‐II/LC3‐I ratio, upregulated Beclin1 and accumulated p62 in the MCT‐PH group, all of which were partially rescued upon Apelin‐13 administration (Figure 1D, Figure S1R), confirming that Apelin‐13 suppresses excessive pathological autophagy in pulmonary arterial hypertension.

Mechanistic assays further demonstrated that Apelin‐13 restored the abundance of anti‐ferroptotic factors, including FSP1 and the small‐molecule cofactor Coenzyme Q10 (Figure 1E,G,I, Figure S1N–Q). Meanwhile, Apelin‐13 modulated both mRNA and protein levels of iron metabolic regulators FTH1 and hepcidin to restore intracellular iron homeostasis. The MCT‐PH group exhibited canonical ferroptotic hallmarks including iron overload, excessive lipid peroxidation and disrupted antioxidant defence, whereas Apelin‐13 treatment effectively reversed these phenotypes (Figure 1H).

Collectively, Apelin‐13 alleviates MCT‐induced pulmonary hypertensive injury by restraining aberrant autophagy, restoring FSP1 abundance and mitigating endothelial ferroptosis. To further define the direct causal relationship among MCT insult, autophagy fluctuation and ferroptosis at the single‐cell level, we established an MCTP‐challenged primary pulmonary arterial endothelial cell model for subsequent cellular mechanistic exploration.

### 3.2 Pharmacological autophagy inhibition by chloroquine reverses MCTP‐induced FSP1 downregulation, ferroptosis activation and pulmonary arterial endothelial dysfunction

We treated primary pulmonary arterial endothelial cells (PAECs) with MCTP over a 0–24 h time course to dynamically monitor changes in autophagy activity, iron homeostasis regulators and ferroptotic phenotypes, laying a temporal foundation for subsequent pharmacological validation. Western blot quantification showed that prolonged MCTP exposure gradually elevated phosphorylated ULK1 (p‐ULK1), Beclin1 abundance and the LC3‐II/LC3‐I ratio in a time‐dependent manner. Meanwhile, the iron exporter FPN and iron storage protein FTH1 continuously declined, while hepcidin was progressively upregulated, indicating progressive autophagy flux activation and disrupted iron balance (Figure S2A,C).

C11‐BODIPY 581/591 dual immunofluorescence staining revealed steadily rising fluorescence intensity of oxidized lipid probes from 0 to 24 h after MCTP treatment, reflecting progressive lipid ROS accumulation (Figure S2B,D). Consistently, biochemical assays detected time‐dependent intracellular ferrous overload, elevated MDA levels and gradual GSH depletion, demonstrating stepwise aggravation of ferroptotic injury (Figure S2E). Collectively, these time‐series experiments confirmed MCTP triggers time‐dependent autophagy activation synchronized with iron dyshomeostasis and progressive ferroptosis in PAECs, yet these correlative data cannot establish direct causal relationships among autophagy, FSP1 and ferroptosis.

We next set three experimental groups (Control, MCTP, MCTP+CQ) to clarify whether pharmacological autophagy blockade reverses MCTP‐driven FSP1 depletion, ferroptosis initiation and endothelial dysfunction. CFDA and CCK‐8 assays demonstrated that MCTP markedly reduced PAEC viability, whereas co‐treatment with chloroquine (CQ) partially rescued cell survival (Figure 2A,B). DCFH‐DA and C11‐BODIPY staining showed massive accumulation of total ROS and lipid peroxides in MCTP‐treated cells, which was effectively alleviated by CQ intervention (Figure 2C,D).

**FIGURE 2:**
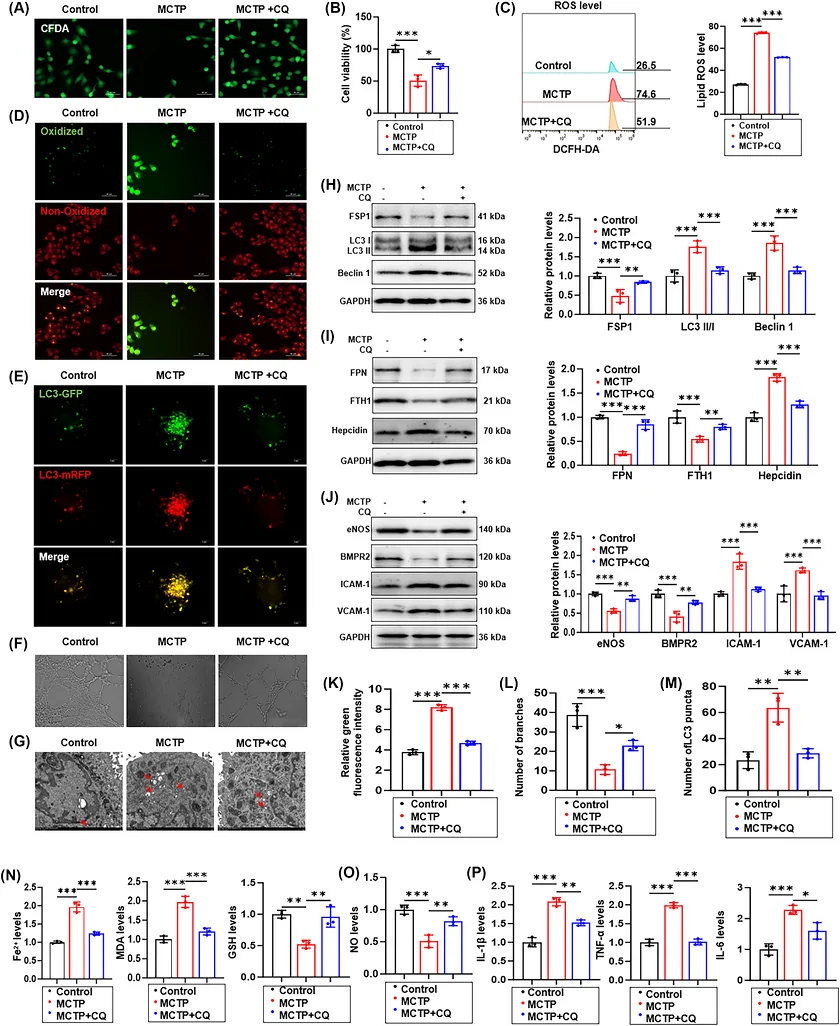
Pharmacological inhibition of autophagy by chloroquine reverses MCTP‐induced FSP1 downregulation, ferroptosis activation and pulmonary arterial endothelial dysfunction. Three experimental groups were set: Control, MCTP, MCTP+CQ (chloroquine). (A and B) CFDA staining and CCK‐8 assay detecting cellular viability of PAECs under different treatments. (C) DCFH‐DA total ROS detection and quantitative analysis of lipid ROS level. (D) Representative C11‐BODIPY immunofluorescence staining for lipid peroxidation. (E, G, M) LC3‐GFP/mRFP tandem fluorescence reporter and transmission electron microscopy monitoring autophagy flux status; quantification of autophagic puncta number. (F and L) Matrigel tube formation assay evaluating endothelial angiogenic capacity and statistical quantification of branching points. (H) Western blot quantification of FSP1, LC3‐I/LC3‐II and Beclin1 to assess autophagy activity and FSP1 abundance. (I and N) Immunoblot analysis of iron homeostasis regulators FPN, FTH1 and hepcidin, along with biochemical measurement of intracellular ferrous content, MDA and GSH. (J and O) Western blot detection of endothelial protective markers (eNOS, BMPR2) and adhesion molecules (ICAM‐1, VCAM‐1), plus quantitative detection of intracellular NO concentration. (P) ELISA quantification of secreted pro‐inflammatory cytokines IL‐1β, TNF‐α and IL‐6. Data are presented as mean ± SD. Statistical analysis was performed via one‐way ANOVA with Tukey's post‐hoc test; *p < .05, **p < .01, ***p < .001; ns, not significant.

Transmission electron microscopy and LC3‐GFP/mRFP tandem fluorescence were used to track autophagy flux. MCTP increased autophagosomal yellow puncta and autolysosomal red‐only puncta, indicative of accelerated autophagy flux; CQ blocked autophagosome–lysosome fusion, trapped autophagosomes and restrained excessive autophagy progression (Figure 2E,G,M). At the molecular level, MCTP elevated the LC3‐II/LC3‐I ratio and Beclin1 while markedly reducing FSP1 abundance, and CQ treatment reversed aberrant autophagy and restored FSP1 expression (Figure 2H).

For iron homeostasis, MCTP downregulated FPN and FTH1 while upregulating hepcidin to trigger intracellular ferrous overload. CQ normalized the expression pattern of these iron regulatory proteins, reversed ferrous accumulation, lowered MDA concentrations and replenished exhausted GSH pools, thereby interrupting the ferroptotic cascade (Figure 2I,N).

To assess endothelial function, tube formation assays revealed impaired angiogenic capacity and fewer branching points in MCTP‐exposed PAECs, which was partially rescued by CQ (Figure 2F,L). Western blot for endothelial markers showed MCTP suppressed protective eNOS and BMPR2 while upregulating adhesion molecules ICAM‐1 and VCAM‐1, characteristic of endothelial dysfunction and pro‐adhesive phenotypic switching. CQ restored eNOS and BMPR2 expression and downregulated adhesion molecules. Correspondingly, MCTP decreased intracellular NO production, and autophagy inhibition recovered NO bioavailability to sustain endothelial homeostasis (Figure 2J,O). ELISA measurements further showed MCTP induced robust secretion of pro‐inflammatory cytokines IL‐1β, TNF‐α and IL‐6, all of which were markedly suppressed upon CQ‐mediated autophagy inhibition, indicating relieved endothelial inflammatory injury (Figure 2P).

In summary, cellular intervention experiments verified that MCTP‐induced excessive autophagy triggers FSP1 loss and subsequent ferroptosis, ultimately reducing PAEC viability and disrupting endothelial function; pharmacological blockade of autophagy by CQ reverses this pathological cascade. These results preliminarily confirm that autophagy negatively regulates FSP1 abundance. We further sought to dissect the bidirectional causal relationship linking autophagy, FSP1 and ferroptosis in subsequent experiments.

### 3.3 Pharmacological autophagy activation exacerbates MCTP‐induced FSP1 depletion and ferroptotic injury in pulmonary arterial endothelial cells

To validate our core hypothesis that autophagy acts as an upstream trigger to degrade FSP1 and initiate ferroptosis, while ferroptosis exerts no reciprocal regulation on autophagy signalling, we treated PAECs with rapamycin (RAPA, a canonical mTOR inhibitor and autophagy agonist) with or without MCTP stimulation, and systematically examined alterations in autophagy flux, FSP1 expression, iron homeostasis and ferroptotic phenotypes. Cell viability assays showed that RAPA further reduced the survival rate of MCTP‐challenged PAECs (Figure S3D).

Transmission electron microscopy and LC3‐GFP/mRFP fluorescence assays revealed that combined MCTP and RAPA treatment produced more autophagosomes and autolysosomes than MCTP alone, confirming accelerated autophagy flux upon RAPA administration (Figure 3A,D, Figure S3G). Quantitative western blot further verified elevated p‐ULK1, Beclin1 and LC3‐II/LC3‐I ratio following RAPA treatment, indicating successful pharmacological upregulation of endogenous autophagy (Figure 3E, Figure S3H). For ferroptotic phenotypes, DCFH‐DA and C11‐BODIPY staining demonstrated that RAPA‐driven autophagy activation aggravated intracellular total ROS and lipid ROS accumulation triggered by MCTP (Figure 3B, Figure S3A,F). Biochemical quantification of ferroptotic markers revealed higher ferrous ion concentration, elevated MDA and aggravated GSH depletion in the MCTP+RAPA group, indicative of markedly exacerbated ferroptosis (Figure 3G). Consistently, RAPA co‐treatment intensified MCTP‐mediated FSP1 downregulation; it further decreased iron exporter FPN and storage protein FTH1 while upregulating hepcidin, leading to more severe disruption of intracellular iron homeostasis (Figure 3F, Figure S3J).

**FIGURE 3:**
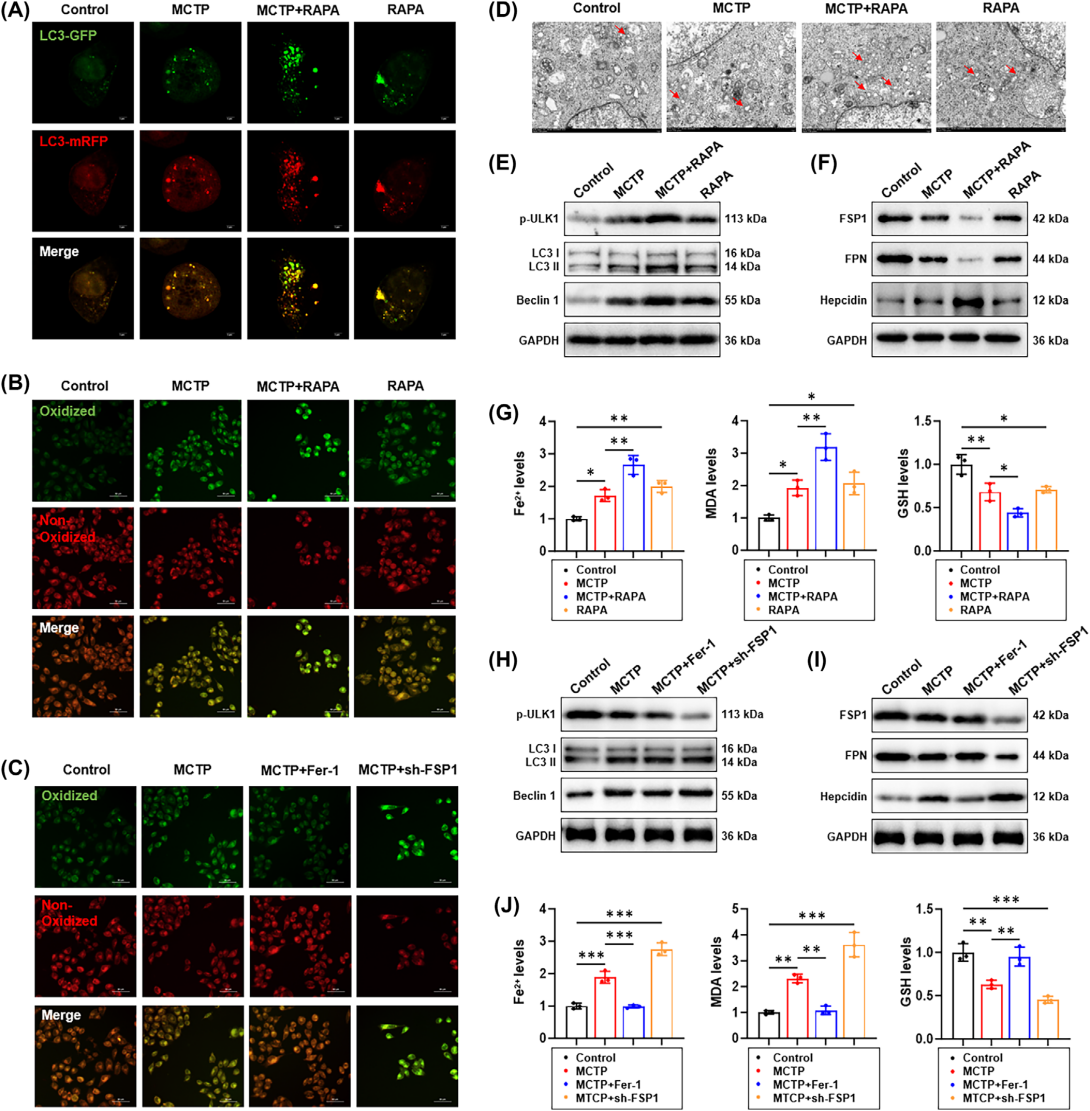
Pharmacological autophagy activation aggravates MCTP‐triggered FSP1 loss and ferroptosis, while ferroptosis exhibits no feedback regulation on autophagy machinery in pulmonary arterial endothelial cells. Two sets of interventions were performed: RAPA‐based autophagy activation (Control, MCTP, MCTP+RAPA, RAPA), and ferroptosis intervention (Control, MCTP, MCTP+Fer‐1, MCTP+sh‐FSP1). (A and D) LC3‐GFP/mRFP tandem fluorescence and transmission electron microscopy to visualize autophagy flux upon RAPA administration. (B) Representative C11‐BODIPY staining detecting lipid peroxidation in RAPA‐treated groups. (C) Representative C11‐BODIPY staining evaluating lipid peroxidation following Fer‐1 or sh‐FSP1 treatment. (E) Western blot detection of autophagy‐related proteins p‐ULK1, LC3‐I/LC3‐II and Beclin1 in RAPA‐intervened cells, with GAPDH as loading control. (F) Immunoblot analysis of FSP1, FPN and hepcidin for iron homeostasis assessment in RAPA‐treated groups. (G) Biochemical quantification of intracellular ferrous ion, MDA and GSH levels reflecting ferroptotic severity in RAPA intervention cohort. (H) Western blot detection of core autophagy proteins after Fer‐1 or sh‐FSP1 treatment. (I) Immunoblot detection of FSP1, FPN and hepcidin in Fer‐1/sh‐FSP1 intervention groups. (J) Biochemical measurement of ferrous ion, MDA and GSH contents to quantify ferroptotic changes upon Fer‐1 or FSP1 knockdown. Data are presented as mean ± SD. Statistical analysis was performed by one‐way ANOVA with Tukey's post‐hoc test; *p < .05, **p < .01, ***p < .001; ns, not significant.

To probe a potential feedback loop between ferroptosis and autophagy, we applied the ferroptosis inhibitor Fer‐1 or shRNA‐mediated FSP1 knockdown to test whether inhibiting ferroptosis execution or FSP1 depletion modulates autophagy machinery. Four groups were set: Control, MCTP, MCTP+Fer‐1, MCTP+sh‐FSP1. Cell viability measurements showed Fer‐1 partially rescued MCTP‐elicited cell death, whereas FSP1 knockdown further reduced PAEC survival (Figure S3C). sh‐FSP1 amplified MCTP‐induced lipid peroxidation, ferrous overload, excessive MDA production and GSH exhaustion to collapse cellular antioxidant defence. In contrast, Fer‐1 markedly eliminated lipid peroxidation and reversed ferroptotic biochemical abnormalities without restoring total FSP1 protein abundance (Figure 3C,I–J, Figure S3B,E,K). Western blot probing core autophagy molecules confirmed neither Fer‐1 nor sh‐FSP1 altered autophagy pathway activity (ns, Figure 3H, Figure S3I).

In conclusion, hyperactivated autophagy functions upstream to reduce FSP1 abundance, disrupt iron homeostasis and trigger ferroptotic cell death. Under these experimental conditions, ferroptosis execution or FSP1 depletion does not appear to feedback‐regulate core autophagy machinery markers in PAECs. This finding eliminates misleading correlative inference, firmly identifies FSP1 as an indispensable intermediate hub linking autophagy and ferroptosis, and establishes a clear unidirectional molecular cascade for subsequent dissection of the precise pathway governing autophagy‐dependent FSP1 degradation.

### 3.4 NBR1 transports FSP1 to autolysosomes for autophagic degradation

We next investigated how autophagy activation regulates FSP1 expression in rat primary PAECs. Autophagy induced by SFM/LG/EBSS treatment led to a decrease in FSP1 protein (Figure 4A, Figure S4A,B), but not mRNA levels (Figure 4B). This downregulation was reversed by autophagy inhibitors (Figure 4C, Figure S4D–F). Consistent with this, CHX chase assays demonstrated that autophagy activation accelerates FSP1 degradation, while autophagy inhibition stabilizes the protein (Figure 4D,E). Collectively, these findings demonstrate that autophagy activation downregulates FSP1 expression by promoting its proteolytic degradation.

**FIGURE 4:**
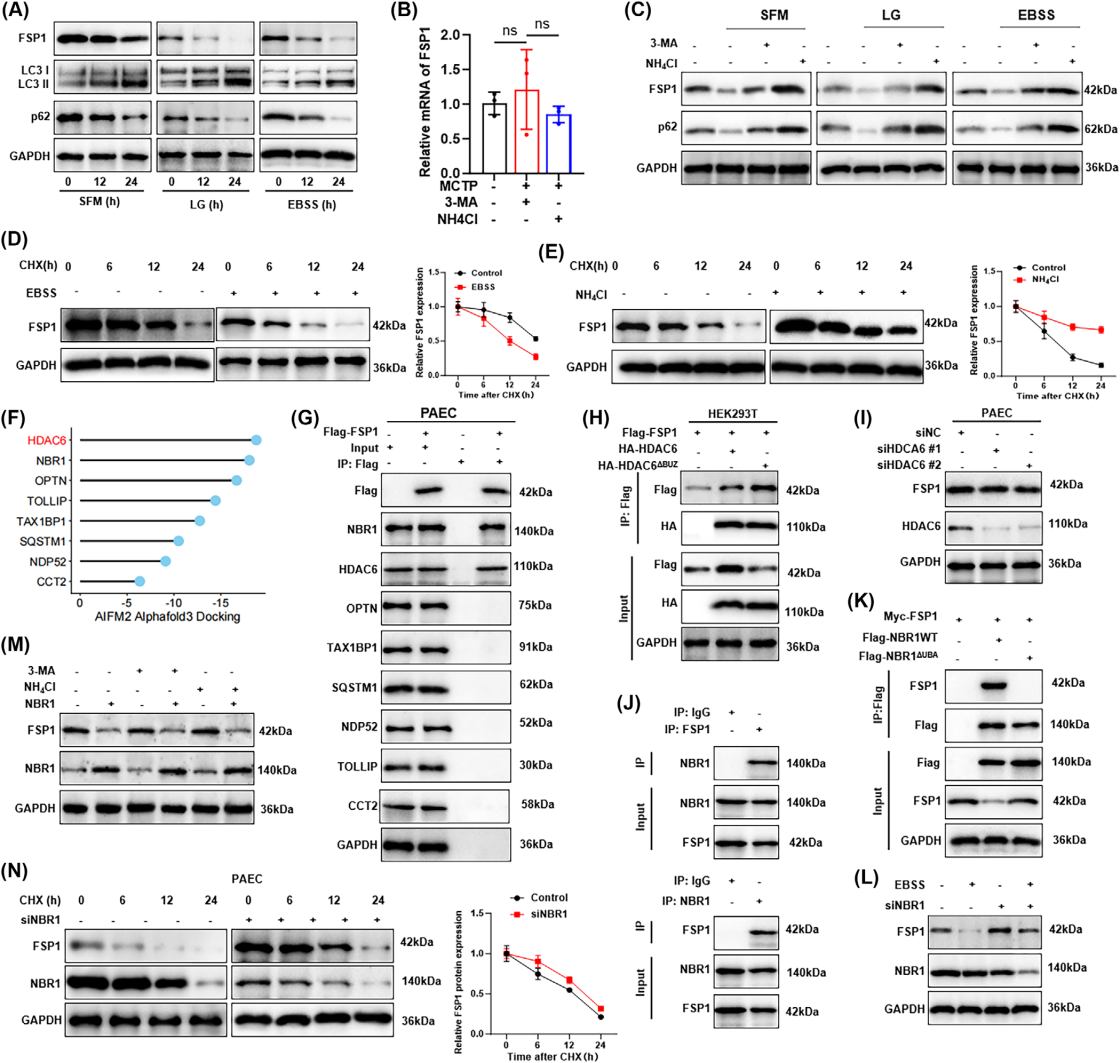
NBR1 mediates autophagic degradation of FSP1 upon autophagy activation. (A) Western blot analysis of FSP1, LC3B, p62 protein expression in PAEC cells subjected to SFM, LG, or EBSS for the indicated durations (0, 12 and 24 h). (B) Analysis of FSP1 mRNA expression levels in MCT‐treated PAEC cells with 3‐MA or NH_4_Cl treatment. (C) Western blot analysis of FSP1, p62 protein expression in PAEC cells under different treatment conditions, with or without treatment with the autophagy inhibitor 3‐MA or NH_4_Cl. (D) Immunoblot analysis was performed using lysates from PAEC cells treated with or without EBSS and CHX (10 µg/mL) at different time points. (E) Immunoblot analysis was performed using lysates from PAEC cells treated with or without NH_4_Cl and CHX (10 µg/mL) at different time points. (F) Screening of potential ALFM2‐interacting proteins by Alphafold3 docking. (G) Co‐IP assay detects interaction between FSP1 and eight selective autophagy receptors. (H) Co‐IP assays were performed in HEK‐293T cells co‐transfected with plasmids encoding Flag‐FSP1, HA‐HDAC6 or HA‐HDAC6^ΔUBD^. (I) Western blot analysis of FSP1 and HDAC6 expression in PAECs after HDAC6 knockdown. (J) Co‐IP assay reveals specific interaction between FSP1 and NBR1. (K) Co‐IP assays were performed in HEK‐293T cells co‐transfected with plasmids encoding Flag‐FSP1, HA‐NBR1 or HA‐NBR1^ΔUBD^. (L) Western blot analysis of FSP1 and NBR1 expression in EBSS‐treated PAECs after NBR1 knockdown. (M) Western blot analysis of FSP1 and NBR1 expression under 3‐MA, NH_4_CI and NBR1 overexpression treatment of PAEC cells. (N) Immunoblot analysis was performed using lysates from PAEC cells treated with or without siNBR1 and CHX (10 µg/mL) at different time points. Statistical analysis was performed by one‐way ANOVA with Tukey's post‐hoc test; *P < 0.05, **P < 0.01, ***P < 0.001; ns, not significant.

To determine whether FSP1 is a substrate for selective autophagic degradation, we screened potential autophagy receptors that might mediate this process. Molecular docking analysis of the binding energy between eight autophagy receptors and FSP1 revealed that HDAC6 and NBR1 had the highest docking scores with FSP1 (Figure 4F). Co‐IP assays confirmed endogenous interactions between both HDAC6/NBR1 and FSP1 in PAECs (Figure 4G). Further validation in HEK‐293T cells showed that mutation of the ubiquitin‐binding domain in HDAC6 abolished its interaction with FSP1, and knockdown of HDAC6 did not affect FSP1 protein expression (Figure 4H–I). In contrast, NBR1 not only exhibited an exogenous interaction with FSP1 (Figure 4J), but this interaction was disrupted by mutation of its key binding site (Figure 4K). Functional assays demonstrated that knockdown of NBR1 significantly suppressed autophagy‐mediated downregulation of FSP1 protein (Figure 4L) and enhanced FSP1 protein stability (Figure 4N), whereas overexpression of NBR1 reversed the upregulation of FSP1 induced by autophagy inhibitors (Figure 4 M).

In summary, autophagy activation mediates the recognition and degradation of FSP1 protein via the autophagy receptor NBR1, thereby downregulating FSP1 expression at the protein level. Mounting evidence has demonstrated that selective autophagy rarely recognizes target proteins directly without prior ubiquitination. Most autophagic substrates require prior ubiquitination catalysed by E3 ubiquitin ligases to generate ubiquitin tags, which are subsequently bound by autophagy receptors such as NBR1. Accordingly, we further screened and identified the key E3 ubiquitin ligase responsible for FSP1 ubiquitination.

### 3.5 The E3 ubiquitin ligase RNFT1 mediates the ubiquitination and degradation of FSP1

Given that selective autophagy often targets substrates of E3 ubiquitin ligases, and considering that NBR1 knockdown only partially stabilized FSP1, we hypothesized that an E3 ligase cooperates in FSP1 degradation. To identify E3 ubiquitin ligases potentially mediating FSP1 degradation, we integrated multi‐omics data using the following criteria: E3 ligases upregulated in PAH (GSE113439), negatively correlated with FSP1 in autophagy‐high samples (GSE254617), and positively associated with the ferroptosis pathway (GSE117261). This analysis identified nine candidate molecules (Figure 5A, Figure S5A–D). RNFT1 showed the highest binding energy with FSP1 in molecular docking (Figure 5B) and was significantly upregulated in both PAH and autophagy‐high tissues (Figure 5C,D). Co‐IP confirmed a specific interaction exclusively between FSP1 and RNFT1 (Figure 5E), with no binding observed to other E3 ligases like OSTM1 or HUWE1 (Figure S5E–I).

**FIGURE 5:**
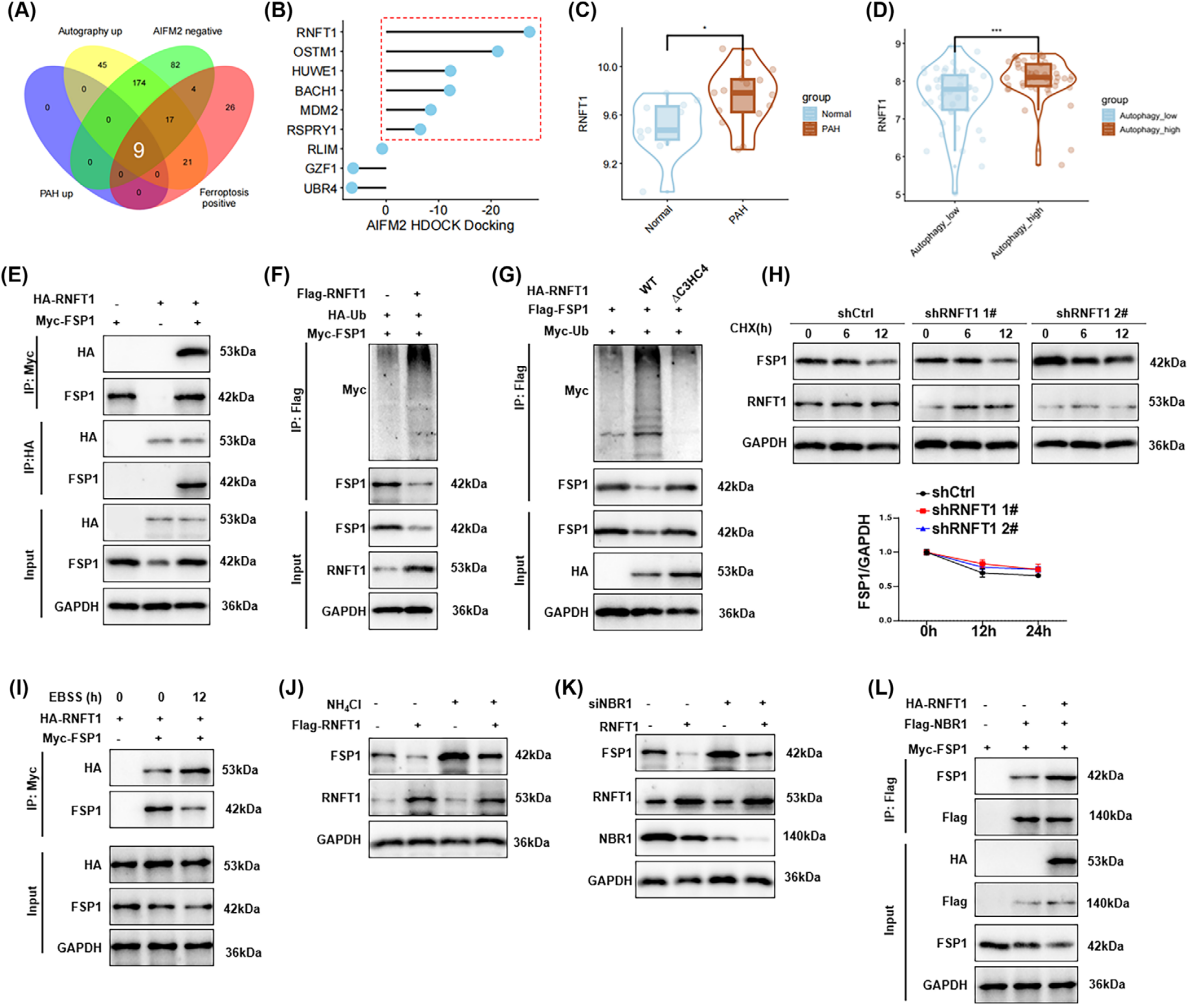
The E3 ubiquitin ligase RNFT1 regulates FSP1 protein expression through ubiquitination‐mediated degradation. (A) Bioinformatic screening of E3 ubiquitin ligases associated with ferroptosis and PAH. (B) The binding affinity between AIFM2 and a panel of target proteins was predicted using the HDOCK molecular docking platform. (C) RNFT1 expression is significantly elevated in PAH patients compared to normal controls. (D) High autophagy levels are associated with increased RNFT1 expression. (E) Co‐IP assay detects Interaction between RNFT1 and FSP1. (F and G) HEK293T cells were co‐transfected with HA‐RNFT1, Myc‐Ub, Flag‐FSP1 (WT and ΔC3HC4), Co‐IP analysis autophagic lysosomal pathway degradation of FSP1. (H) PAEC cells were treated with or without RNFT1 shRNA, Western blot analysis protein degradation of SLC40A1. (I) HEK293T cells were transfected with Myc‐FSP1, HA‐RNFT1 and EBSS treatment, Co‐IP analysis the interaction between FSP1 and RNFT1. (J) PAEC cells were transfected with Flag‐RNFT1 and NH_4_CI treatment, Western blot analysis protein expression of FSP1 and RNFT1. (K) PAEC cells were transfected with siNBR1 and RNFT1 treatment, Western blot analysis protein expression of FSP1, RNFT1 and NBR1. (L) HEK293T cells were transfected with Myc‐FSP1, HA‐RNFT1 and Flag‐NBR1, Co‐IP analysis the interaction between FSP1 and RNFT1. Statistical analysis was performed by one‐way ANOVA with Tukey's post‐hoc test; *p < .05, **p < .01, ***p < .001; ns, not significant.

To determine whether RNFT1 mediates the ubiquitination and degradation of FSP1, we co‐overexpressed HA‐RNFT1 (or its catalytically inactive mutant ΔC3HC4), Myc‐FSP1 and HA‐Ubiquitin in HEK293T cells, followed by Co‐IP using a Flag antibody. The results demonstrated that RNFT1 promotes FSP1 ubiquitination in an E3 ligase activity‐dependent manner (Figure 5F,G). CHX chase assays indicated that RNFT1 knockdown enhances FSP1 protein stability (Figure 5H). Furthermore, autophagy activation strengthened the FSP1‐RNFT1 interaction (Figure 5I), while RNFT1 overexpression attenuated the accumulation of FSP1 induced by autophagy inhibition (Figure 5J), suggesting that autophagy activation may cooperatively regulate FSP1 levels by facilitating RNFT1‐mediated ubiquitination and degradation. Further experiments showed that RNFT1 overexpression reversed FSP1 accumulation caused by NBR1 knockdown, while simultaneously enhancing FSP1 binding to NBR1 (Figure 5K,L). These results demonstrate that RNFT1‐mediated ubiquitination and NBR1‐mediated selective autophagy synergistically regulate FSP1 degradation.

### 3.6 Chloroquine attenuates MCT‐induced PAEC injury by stabilizing FSP1

Given the association between FSP1 deficiency and autophagy activation, we further investigated the impact of autophagy inhibitors on FSP1 protein stability. Results showed that while treatment with varying concentrations of CQ did not affect FSP1 mRNA levels (Figure 6A,B,H), it significantly upregulated FSP1 protein expression (Figure 6A,B). Mechanistically, CQ enhanced FSP1 stability by inhibiting its K63‐linked polyubiquitination and subsequent proteasomal degradation (Figure 6C). Furthermore, CQ counteracted the NBR1 overexpression‐mediated downregulation of FSP1 protein (Figure 6D), while combined inhibition of NBR1 and CQ treatment synergistically restored FSP1 protein levels (Figure 6E). Exogenous co‐transfection experiments further confirmed that CQ treatment reduced the amount of FSP1 bound to NBR1 (Figure 6F). Finally, CQ treatment at different concentrations effectively restored FSP1 protein expression and ameliorated corresponding iron metabolism dysregulation (Figure 6G,I).

**FIGURE 6:**
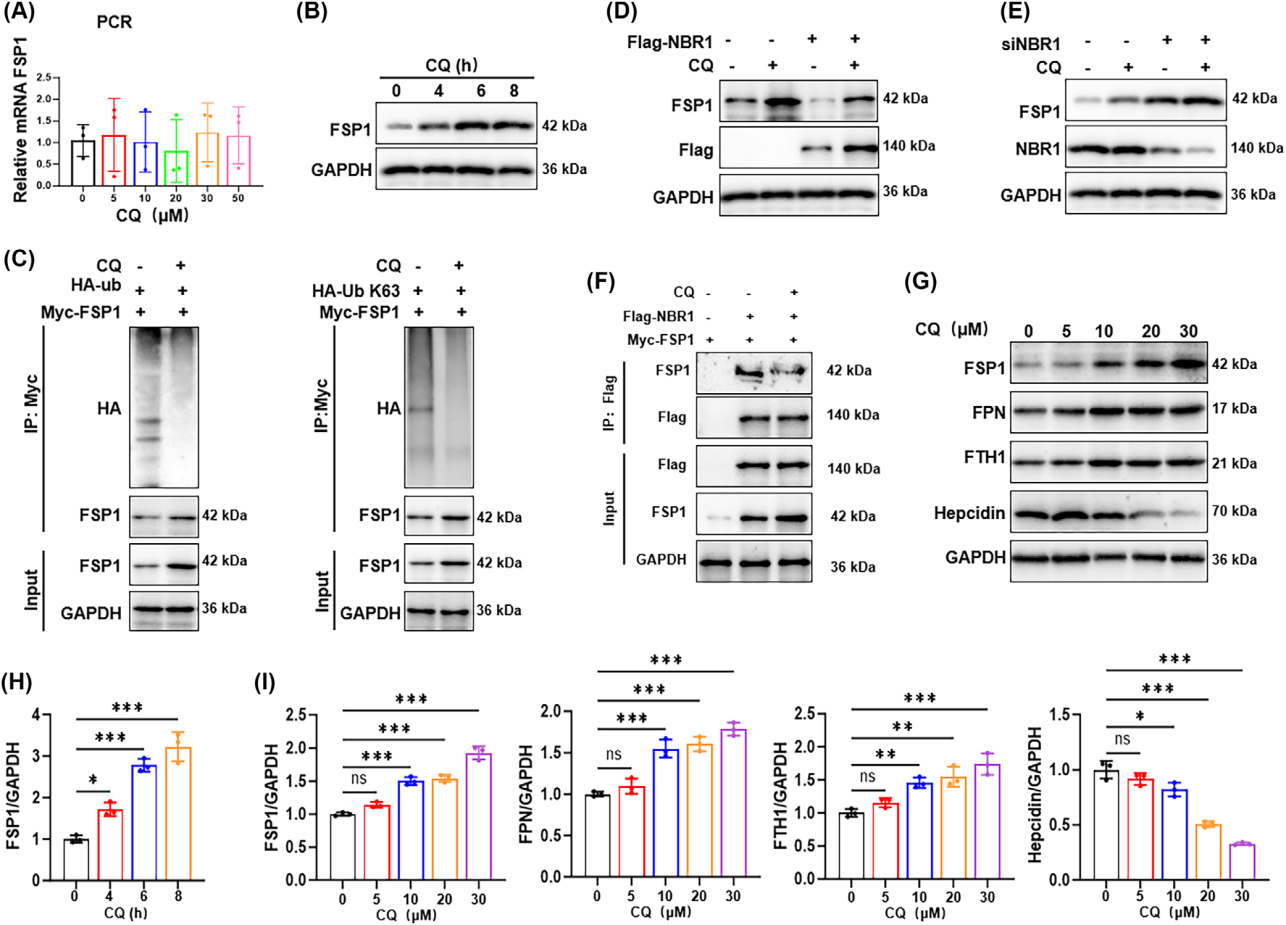
Chloroquine stabilizes FSP1 protein by suppressing its K63‐linked ubiquitination and NBR1‐dependent autophagic degradation. (A) Quantitative analysis of FSP1 mRNA expression in response to increasing concentrations of CQ. (B) Western blot analysis of FSP1 protein expression upon CQ treatment. C. HEK‐293T cells were transfected with Myc‐FSP1, HA‐Ub or HA‐Ub K63, Co‐IP detection of autolysosomal degradation of FSP1 after CQ treatment. (D) Western blot analysis of FSP1, Flag‐NBR1 and GAPDH expression in rat PAECs transfected with Flag‐NBR1, with or without CQ treatment. (E) Western blot analysis of FSP1, NBR1 and GAPDH expression in rat PAECs transfected with siNBR1, with or without CQ treatment. (F) Effect of CQ treatment on the interaction between FSP1 and NBR1 in HEK293T cells. (G) Western blot of FSP1, FPN, FTH1, Hepcidin and GAPDH expression in different CQ treatment. (H) Quantitative analysis of FSP1 protein expression upon CQ treatment. (I) Quantitative analysis of FSP1, FPN, FTH1, Hepcidin expression in different CQ treatment. Statistical analysis was performed by one‐way ANOVA with Tukey's post‐hoc test; *p < .05, **p < .01, ***p < .001; ns, not significant.

### 3.7 Combination therapy of FSP1 overexpression and Apelin‐13 alleviates the progression of PAH in rats

Existing evidence confirms autophagy promotes FSP1 degradation to initiate endothelial ferroptosis and drive PAH pathogenesis, whereas Apelin‐13 only indirectly stabilizes endogenous FSP1 via autophagy suppression. We hypothesized that AAV‐mediated FSP1 overexpression combined with Apelin‐13 exerts synergistic therapeutic benefits by directly replenishing FSP1 and simultaneously blocking its autophagic degradation. Three experimental groups were established for phenotypic, molecular and safety validation: MCT‐PH, MCT‐PH+AAV‐FSP1 and MCT‐PH+AAV‐FSP1+Apelin‐13.

AAV‐FSP1 monotherapy partially alleviated pulmonary arteriolar medial thickening, collagen deposition, aberrant smooth muscle proliferation and endothelial injury, while combination treatment reversed vascular remodelling and secondary right ventricular hypertrophy to a more pronounced degree (Figure 7A, Figure S6A). Consistently, AAV‐FSP1 moderately normalized haemodynamic parameters (mPAP, PVR, RVSP, RVEDP), restored body weight gain, prolonged pulmonary acceleration time and partially rescued cardiac output to relieve chronic pressure overload‐induced right heart dysfunction (Figure 7B–D). The combinatorial regimen generated superior alleviation of pulmonary lesions, more effective normalization of pulmonary circulatory overload and greater recovery of cardiac output.

**FIGURE 7:**
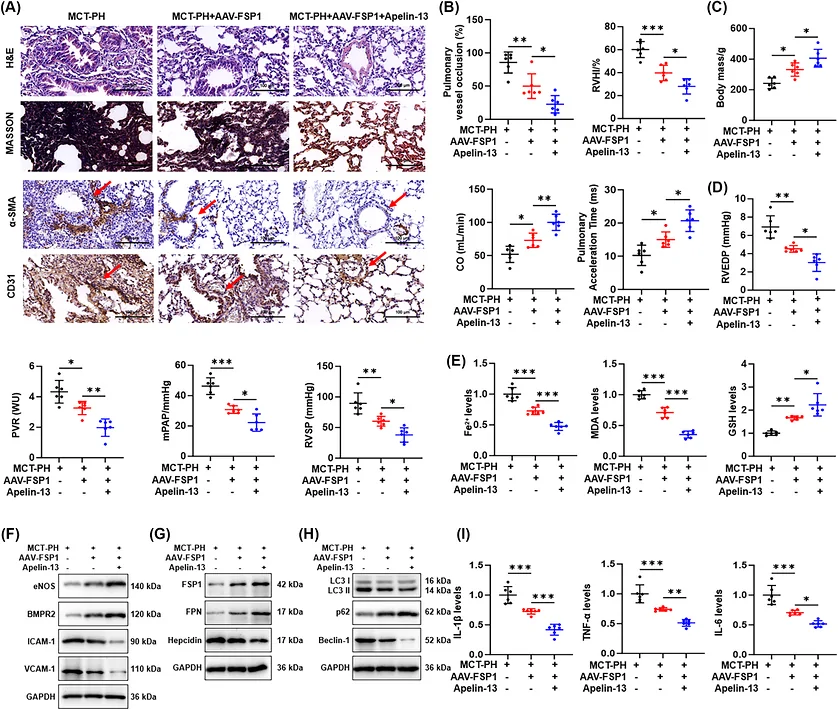
Combined AAV‐mediated FSP1 overexpression and Apelin‐13 administration exerts synergistic protective effects against monocrotaline‐induced pulmonary arterial hypertension in vivo. Three experimental groups: MCT‐PH, MCT‐PH+AAV‐FSP1, MCT‐PH+AAV‐FSP1+Apelin‐13. (A) Representative H&E and Masson's trichrome staining of pulmonary arterioles; immunohistochemical staining for α‐SMA and CD31 to evaluate vascular remodelling and endothelial integrity. (B–D) Quantitative statistics of pulmonary vessel occlusion rate, right ventricular hypertrophy index, body weight, pulmonary acceleration time, RVEDP, PVR, mPAP, RVSP and cardiac output for haemodynamic and cardiac functional assessment. (E) Biochemical quantification of intracellular ferrous ion, MDA and GSH levels to assess ferroptotic severity. (F) Western blot analysis of endothelial functional proteins eNOS, BMPR2, ICAM‐1 and VCAM‐1, with GAPDH as internal reference. (G) Immunoblot detection of FSP1, FPN and hepcidin for iron homeostasis evaluation. (H) Western blot measurement of autophagy‐related proteins LC3‐I/LC3‐II, p62 and Beclin‐1. (I) Quantitative analysis of pro‐inflammatory cytokines IL‐1β, TNF‐α and IL‐6. Data are presented as mean ± SD. Statistical analysis was performed by one‐way ANOVA with Tukey's post‐hoc test; *p < .05, **p < .01, ***p < .001; ns, not significant.

At the molecular level, AAV‐FSP1 moderately elevated FSP1 abundance, corrected iron dyshomeostasis, upregulated core anti‐ferroptotic proteins including FSP1 and coenzyme Q10, and mitigated canonical ferroptotic hallmarks such as ferrous overload, MDA accumulation and GSH depletion (Figure 7E,G, Figure S6B,D). Combination treatment further upregulated FSP1 and robustly reversed iron overload, lipid peroxidation and antioxidant system collapse. Although AAV‐FSP1 alone exerted mild inhibitory effects on excessive autophagy (Figure 7H, Figure S6C), it rescued endothelial protective eNOS and BMPR2, downregulated adhesion molecules ICAM‐1/VCAM‐1 (Figure 7F, Figure S6E), and alleviated vascular inflammation mediated by IL‐1β, TNF‐α and IL‐6 (Figure 7I). These protective phenotypes were further strengthened under combined intervention. Systemic safety assessment verified that neither treatment induced hepatic, splenic or renal toxicity, with stable body weight and normal hepatic–renal biochemical indexes across all groups (Figure S6F–H).

In summary, AAV‐FSP1 monotherapy partially attenuates MCT‐induced PAH, whereas its combination with Apelin‐13 produces synergistic protection. This dual strategy stabilizes FSP1 through exogenous overexpression and autophagy‐mediated anti‐degradation, potently suppresses endothelial ferroptosis, reverses pathological vascular remodelling, rectifies haemodynamic dysfunction and alleviates inflammatory injury with favourable systemic biocompatibility, serving as a promising combinatorial candidate for translational PAH therapy.

## 4 DISCUSSION

This study identifies FSP1 as a key molecule linking autophagy and ferroptosis in PAH, whose protein stability is regulated by a precise network involving NBR1‐mediated autolysosomal degradation and RNFT1‐mediated ubiquitination. Based on this mechanism, the combination therapy of FSP1 overexpression and Apelin‐13 demonstrated potent synergistic efficacy in vivo, significantly delaying PAH progression. Our work not only deepens the understanding of PAH pathogenesis but also establishes a novel therapeutic concept targeting FSP1 degradation.

Previous research on PAH has primarily focused on the roles of ferroptosis or autophagy as independent pathogenic pathways.^29^, ^30^ Although these studies have provided valuable insights, this unilateral perspective offers a limited understanding of the integrated pathogenic network driving PAH progression.^31^ Our study significantly advances the field by not only establishing a strong correlation between autophagy and ferroptosis in PAH but also identifying FSP1 as the central molecular node connecting these two critical processes. We further elucidate a sophisticated regulatory network in which FSP1 protein stability is coregulated by dual degradation pathways. This discovery shifts the research paradigm from viewing autophagy and ferroptosis as parallel events to understanding their direct crosstalk via specific protein nodes, providing a novel and more comprehensive perspective on the pathogenesis of PAH.

Beyond the established post‐translational regulation of FSP1, our data intriguingly indicate that Apelin‐13 upregulates *FSP1* mRNA in PAH rats,^32^, ^33^ suggesting potential transcriptional‐level dysregulation in vivo, which is complemented by our primary mechanistic findings on FSP1 degradation. Furthermore, while this study focused on pulmonary arterial endothelial cells (PAECs), the cell‐autonomous role of FSP1 in other pivotal vascular cells, particularly pulmonary arterial smooth muscle cells (PASMCs), remains unexplored.^34^ Future investigations into these cell‐specific functions and transcriptional mechanisms are warranted. Notably, our causal inference relies solely on pharmacological autophagy modulators (CQ, rapamycin), which carry inherent off‐target risks independent of autophagy flux; genetic validation via ATG5/ATG7 perturbation will be required to consolidate the causal cascade between autophagy and FSP1 loss. Subsequently, the long‐term efficacy and safety of targeting the FSP1/Apelin‐13 axis must be rigorously evaluated in chronic PAH models to assess its therapeutic potential.

We also acknowledge two competing regulatory layers for FSP1 turnover: first, autophagy‐independent K48‐linked ubiquitin‐proteasome turnover may concurrently modulate FSP1 abundance, which remains uncharacterized herein; second, though NBR1 preferentially binds RNFT1‐deposited K63 ubiquitin chains on FSP1, NBR1 can recognize other ubiquitin topologies in alternative cellular contexts, leaving open whether distinct ubiquitin modifications redirect FSP1 degradation under divergent pathological stimuli. An unexpected observation was that lysosomal inhibitor CQ suppressed FSP1 K63 ubiquitination despite lacking direct E3 ligase inhibitory activity. Two concise explanations are proposed: negative feedback from accumulated stabilized FSP1 competitively inhibits RNFT1 catalytic function, or CQ remodels RNFT1 subcellular localization via pH perturbation to weaken FSP1‐RNFT1 binding, independent of autophagy blockade.

Based on the FSP1‐NBR1‐RNFT1 regulatory axis identified in this study, targeted drug screening and candidate molecule discovery are highly feasible. The key protein structure of this pathway is well‐defined, and its regulatory mechanism is clearly understood, making it an excellent target for rational drug design. Utilizing modern strategies such as computer‐aided drug design and high‐throughput screening, novel small molecule compounds that stabilize FSP1, inhibit NBR1 recognition, or block the activity of RNFT1 ubiquitin ligase can be rapidly identified.^35^, ^36^ Targeting the CoQ10 binding pocket of FSP1 or its interaction interface with NBR1/RNFT1 through structure‐based virtual screening and molecular docking allows for the rapid identification of small molecule precursor compounds that can directly stabilize FSP1, obstruct NBR1 recognition, or inhibit the activity of the RNFT1 ubiquitin ligase. As evidenced by the skeleton design and structure‐activity relationship (SAR) studies on kinases such as CDK8 and EGFR, rational optimization of interactions with key residues in the binding pocket can significantly enhance the selectivity and inhibitory activity of compounds, thereby establishing a robust paradigm for the development of new therapeutics for pulmonary arterial hypertension.^37^, ^38^ RNFT1, a critical E3 ubiquitin ligase, has a catalytic domain that can serve as a target for small molecule inhibitors. By inhibiting its E3 ligase activity, it can prevent ubiquitination at the K63 site of FSP1 and its subsequent degradation.^39^ Concurrently, small molecules that disrupt the binding of NBR1 and FSP1 can also be developed to impede their autophagic degradation. This dual‐target inhibition strategy can synergistically stabilize FSP1 protein levels and exert a more pronounced inhibitory effect on ferroptosis.^40^

The Apelin‐13 combined FSP1 activation strategy confers prominent clinical translational value: it stabilizes FSP1 through dual layers of intervention (blocking ubiquitin‐autophagic degradation and supplementing exogenous functional FSP1), resolving the insufficient therapeutic efficacy of single‐target drugs currently used for PAH. This targeted regimen is broadly applicable to multiple PAH subtypes, including idiopathic PAH, chronic thromboembolic PAH and fibrosis‐complicated PAH, all of which exhibit FSP1 depletion and endothelial ferroptosis. As a low‐toxicity endogenous peptide, Apelin‐13 has long‐acting modified analogues undergoing cardiovascular preclinical research, substantially lowering the clinical translation threshold. Moreover, this therapeutic paradigm can be extended to other cardiovascular and fibrotic diseases with ferroptosis‐autophagy disorders, greatly broadening its clinical application scope. Collectively, the FSP1 regulatory axis uncovered in this work represents a promising druggable target and provides an innovative mechanistic basis for causal PAH treatment.

Despite such promising translational prospects, critical clinical bottlenecks restrict its immediate clinical implementation. First, AAV‐mediated FSP1 gene delivery suffers from poor pulmonary endothelial tropism, limiting stable pulmonary vascular transfection efficiency for long‐term clinical use. Second, sustained systemic autophagy inhibition by long‐term Apelin‐13 administration may interfere with homeostatic autophagy in cardiomyocytes and intact endothelial cells, introducing unknown cardiovascular safety risks for chronic clinical medication. Third, FSP1 expression dynamics across varying PAH disease stages and patient subgroups remain unclear, and no circulating peripheral biomarkers are available to screen patients eligible for FSP1‐targeted therapy, posing challenges for patient stratification in future clinical trials. Future translational research must prioritize developing pulmonary endothelium‐specific delivery carriers and selective small‐molecule inhibitors disrupting RNFT1‐NBR1 binding, and verify therapeutic efficacy in humanized large‐animal PAH models to overcome these obstacles and accelerate the clinical translation of FSP1‐centered therapy.

Current drug research and development is experiencing a paradigm shift from occupancy‐driven inhibition to event‐driven degradation, with PROTAC degraders emerging as a focal point of innovation in both academia and industry.^41^, ^42^ For the RNFT1 and NBR1 targets identified in this study, the development of selective PROTAC molecules can precisely regulate the FSP1 degradation pathway, thereby overcoming the limitations associated with traditional small molecule inhibitors. PROTAC technology, characterized by its high selectivity, efficiency and catalytic degradation, demonstrates significant potential for application in various diseases, including cardiovascular conditions and tumors.^43^ By rationally designing linkers and target heads, it is possible to construct PROTAC molecules that specifically target RNFT1 or NBR1, effectively blocking the pathological degradation of FSP1. Established PROTAC development strategies and case studies offer mature technical pathways and theoretical support for the design of innovative drugs targeting the pathways examined in this study.

From a translational perspective, this study successfully bridges fundamental mechanistic discoveries to the development of a viable therapeutic strategy. The combination therapy of FSP1 overexpression and Apelin‐13, which we developed, demonstrated remarkable synergistic efficacy in animal models by concurrently targeting two pivotal pathological processes: vascular function modulation and cytoprotection, thereby effectively attenuating PAH progression.^44^ Our work signifies a paradigm shift in therapeutic intervention, moving from symptomatic management toward mechanism‐based causal therapy. We propose a novel therapeutic paradigm involving exogenous supplementation of functional FSP1 protein coupled with inhibition of its pathological degradation. This multi‐target approach simultaneously acts on several key nodes of the complex PAH pathological network, offering the potential for more durable and fundamental therapeutic outcomes. Thus, our findings not only validate targeting the FSP1 degradation pathway as a feasible treatment for PAH, but more importantly, provide a conceptual framework and solid experimental groundwork for developing next‐generation precision combination therapies against complex diseases.

## 5 CONCLUSION

We demonstrated that FSP1 serves as a critical molecule linking autophagy and ferroptosis, and its downregulation is a key driver of PAH progression (Graphical Abstract). Mechanistically, FSP1 protein stability is coregulated by dual degradation pathways: the autophagy receptor NBR1 recognizes and binds FSP1, shuttling it to autolysosomes for degradation via the LC3‐mediated autophagy pathway; concurrently, the E3 ubiquitin ligase RNFT1 mediates K63‐linked polyubiquitination of FSP1; this ubiquitin tag serves as a recognition signal for autophagy receptor NBR1, ultimately shuttling FSP1 into autolysosomes for lysosomal degradation. Together, these pathways form a sophisticated regulatory network controlling FSP1 stability. Our study not only elucidates a novel mechanism by which autophagy–ubiquitin crosstalk regulates FSP1 stability and consequently influences ferroptosis, but also proposes a new therapeutic concept of targeting FSP1 degradation for PAH treatment, laying a critical theoretical foundation for future translational research. This study elucidates a novel crosstalk mechanism between RNFT1‐mediated K63 ubiquitination and selective autophagy that jointly governs FSP1 protein stability, thereby modulating endothelial ferroptosis and PAH progression. In addition, it proposes a new therapeutic strategy focused on targeting FSP1 degradation for the treatment of pulmonary arterial hypertension.

## AUTHOR CONTRIBUTIONS

All authors confirm that they have made substantial contributions to this work and have approved the final version of the manuscript for submission. Specifically, Lihua Guan and Wenzhi Pan conceived and designed the research; Dandan Chen and Qi Jin performed the experiments; Lei Zhang conducted the data analysis; Dandan Chen drafted the manuscript; Lihua Guan, Daxin Zhou and Junbo Ge took charge of data verification and critical revision of the manuscript for important intellectual content. Lihua Guan serves as the principal corresponding author, who is responsible for all correspondence concerning this manuscript, including addressing reviewer comments, coordinating revisions and responding to post‐publication queries.

## CONFLICT OF INTEREST STATEMENT

The authors declare no conflicts of interest.

## ETHICS APPROVAL

This study was approved by the Ethics committee of Zhongshan Hospital, Fudan University, Shanghai Institute of Cardiovascular Diseases, Shanghai, China.

## Supporting information



Supporting Information

Supporting Information

## Data Availability

The data analysed during the current study are available from the corresponding author on reasonable request.
