# Supplementary material for: FSP1 as the convergent hub of autophagy and ubiquitination governing ferroptosis in monocrotaline‐induced pulmonary arterial hypertension
Source: Clin Transl Med. 2026 Nov 5;16(11):e70822. doi: 10.1002/ctm2.70822 (PMC13736785; doi:10.1002/ctm2.70822)
Supplement: Supplementary file 1 [file CTM2-16-e70822-s001.docx]

**Supplementary Figures**

**Figure S1. Identification of FSP1 as a key downregulated gene linking autophagy -ferroptosis correlation in PAH. A.** Box plot comparing ferroptosis scores between autophagy low and autophagy high groups of samples from the GSE117261 dataset. **B.** Scatter plot showing a significant positive correlation between autophagy and ferroptosis scores. **C.** Volcano plot displaying DEGs between autophagy high and autophagy low groups. AIFM2 is highlighted. **D.** Volcano plot displaying DEGs between PAH and control samples from the GSE117261 dataset. AIFM2 is highlighted. **E.** Venn diagram illustrating the overlap of downregulated genes between the PAH dataset and the autophagy-related dataset. **F-G.** UMAP visualization of lung single-cell populations from healthy donors and PAH patients based on dataset GSE210248, with cell-type annotation. **H-I.** Cellular distribution, average expression and positive proportion statistics of *AIFM2* across all lung cell subtypes. **J-K.** Quantitative comparison of *AIFM2* expression and positive cell percentage in endothelial cells between donor and PAH groups. **L.** Pseudobulk analysis verifying overall decreased *AIFM2* transcription in PAH patients (*P* = 0.04). **M.** Quantitative statistics of pulmonary vascular fibrosis ratio, medial thickness index, cardiac collagen deposition, α-SMA-positive cell proportion and CD31-positive endothelial cell percentage from immunohistochemical staining. **N-O.** Quantitative western blot and qPCR analysis verifying FSP1 protein and mRNA expression changes across three experimental groups. **P-Q.** Relative protein and mRNA quantification of iron metabolic regulators FPN and hepcidin. **R.** Quantitative analysis of autophagy-associated protein expression (LC3-II/LC3-I ratio, p62, Beclin-1). Data are presented as mean ± SD. Statistical differences were analyzed by one-way ANOVA followed by Tukey’s post-hoc test; *P* < 0.05, P < 0.01, *P < 0.001.

**Figure S2 Time-dependent induction of autophagy, disrupted iron homeostasis and progressive ferroptosis in primary pulmonary arterial endothelial cells upon MCTP stimulation**

**A.** Western blot analysis showing dynamic expression of p-ULK1, Beclin1, LC3-I/LC3-II, FPN, FTH1 and hepcidin in PAECs treated with MCTP for 0, 2, 4, 6, 12 and 24 h; GAPDH served as the internal loading control. **B.** Representative C11-BODIPY 581/591 dual immunofluorescence staining detecting lipid peroxidation at indicated time points after MCTP exposure. Oxidized lipids exhibit green fluorescence, while non-oxidized lipids display red fluorescence. **C.** Quantitative statistical analysis for relative protein levels of autophagy and iron-regulatory proteins corresponding to panel (A). **D.** Quantification of relative green fluorescence intensity from C11-BODIPY staining in panel (B). **E.** Biochemical quantification of intracellular ferrous ion concentration, MDA content and GSH levels reflecting progressive ferroptotic injury over the MCTP treatment time course. Data are presented as mean ± SD. Statistical analysis was performed via one-way ANOVA with Tukey’s post-hoc test; *P* < 0.05, P < 0.01, *P < 0.001.

**Figure S3 Pharmacological activation of autophagy exacerbates MCTP-induced FSP1 depletion, ROS accumulation and ferroptotic injury, and interventions targeting ferroptosis do not feedback regulate autophagy activity**

**A, F.** DCFH-DA staining for total intracellular ROS and quantitative analysis of lipid ROS levels in Control, MCTP, MCTP+RAPA and RAPA groups. **B, E.** C11-BODIPY 581/591 immunofluorescence and corresponding lipid ROS quantification in Control, MCTP, MCTP+Fer-1 and MCTP+sh-FSP1 groups. **C, D.** CCK-8 cell viability assay for two independent intervention systems (RAPA treatment group; Fer-1/sh-FSP1 intervention group). **E, G.** Quantitative statistics of relative green fluorescence intensity and LC3 puncta number from autophagy flux staining. **H, I.** Quantified relative protein expression of p-ULK1, LC3-II/LC3-I and Beclin1 in RAPA-treated cohort and Fer-1/sh-FSP1 intervention cohort, respectively. **J,** **K.** Quantitative western blot analysis of FSP1, FPN and hepcidin abundance under two intervention strategies. Data are presented as mean ± SD. Statistical analysis was performed by one-way ANOVA with Tukey’s post-hoc test; P < 0.05, P < 0.01, *P < 0.001; ns, not significant.

**Figure S4 NBR1 mediates autophagic degradation of FSP1 upon autophagy activation.**

**A-C.** Western blot analysis of FSP1, LC3B, p62 protein expression in PAEC cells subjected to SFM, LG, or EBSS for the indicated durations (0, 12, and 24 hours). **D-F.** Western blot analysis of FSP1, p62 protein expression in PAEC cells under different treatment conditions, with or without treatment with the autophagy inhibitor 3-MA or NH₄Cl.

**Fig S5. Identification of FSP1-interacting E3 ubiquitin ligases and their functional characterization in PAH.**

**A-D.** Bioinformatic screening of E3 ubiquitin ligases associated with ferroptosis and PAH. **(A)** Differentially expressed genes in PAH patients. **(B)** Autophagy-related E3 ligases. **(C)** FSP1-negative correlated E3 ligases. **(D)** Ferroptosis-positive associated E3 ligases. **E-I.** Co-IP assay shows no specific interaction between FSP1 and OSTM1**(E)**, HUWE1**(F)**, BACH1**(G)**, MDM2**(H)** and RSPRY1**(I)**.

**FigS6. Combination of AAV-FSP1 and Apelin-13 induces no toxicity in major organs.**

**A.** Quantitative statistics of right ventricular fibrosis ratio, medial thickness index, cardiac collagen deposition, α-SMA-positive cell proportion and CD31-positive endothelial cell percentage from immunohistochemical staining. **B.** Quantification of NADH-CoQ reductase activity and NADP⁺/NADPH redox ratio reflecting anti-ferroptotic capacity. **C.** Relative protein quantification of autophagy-associated proteins LC3-II/LC3-I, p62 and Beclin-1. **D.** Western blot quantitative analysis of FSP1, FPN and hepcidin expression. **E.** Quantitative analysis of endothelial protective proteins (eNOS, BMPR2) and adhesion molecules (ICAM-1, VCAM-1). **F.** Longitudinal body weight monitoring throughout the intervention period. **G.** Representative H&E staining of liver, spleen and kidney tissues for visceral morphological toxicity assessment. **H.** Serum biochemical detection of hepatic and renal function indicators (ALT, AST, ALT/AST, urea, creatinine). Data are presented as mean ± SD. Statistical analysis was performed by one-way ANOVA with Tukey’s post-hoc test; P < 0.05, P < 0.01, *P < 0.001; ns, not significant.
