# Supplementary material for: FSP1 as the convergent hub of autophagy and ubiquitination governing ferroptosis in monocrotaline‐induced pulmonary arterial hypertension
Source: Clin Transl Med. 2026 Nov 5;16(11):e70822. doi: 10.1002/ctm2.70822 (PMC13736785; doi:10.1002/ctm2.70822)
Supplement: Supplementary file 2 [file CTM2-16-e70822-s002.docx]

**Methods**

**Animals**

Four-week-old male Sprague-Dawley (SD) rats weighing 180–220 g were purchased from Shanghai SLAC Laboratory Animal Co., Ltd. (Shanghai, China). All animal feeding, housing and experimental protocols were strictly approved by the Institutional Animal Care and Use Committee (IACUC) of the Institutional Animal Care and Use Committee of Zhongshan Hospital, Fudan University, Shanghai Institute of Cardiovascular Diseases. All rats were raised under standard SPF-grade laboratory animal conditions with a 12 h light/dark cycle, constant temperature (22 ± 2°C) and humidity (50% ± 10%), and free access to standard rodent chow and sterile drinking water.

**Rat PAH Model:** A rat model of pulmonary arterial hypertension (PAH) was induced in 4-week-old male Sprague-Dawley (SD) rats by a single subcutaneous injection of monocrotaline (MCT; 60 mg/kg) or an equivalent volume of solvent. The model was evaluated after 3 weeks.

**Therapeutic Intervention:** For the Apelin-13 intervention group, apelin-13 (10 nmol/kg) was administered daily via intraperitoneal injection for 3 consecutive weeks. For the AAV9-FSP1 viral intervention group, each rat received a total virus dose of 2.5 × 10¹⁵ vector genomes (vg) in 300 μL of PBS. The control and model groups received an equivalent volume of normal saline. Hemodynamic measurements and tissue collection were performed at week 5.

**Cells and Cell Culture**

**Rat Pulmonary Arterial Endothelial Cells (PAECs):** Primary PAECs were isolated from rat lung tissues under sterile conditions. Briefly, the pulmonary artery and its main branches were carefully dissected under a stereomicroscope, followed by enzymatic digestion with a combination of collagenase and elastase to dissociate the vascular tissue into a single-cell suspension. The isolated PAECs were cultured in complete endothelial cell growth medium, consisting of DMEM/F12 supplemented with 20% fetal bovine serum (FBS), 1% penicillin-streptomycin, endothelial cell growth supplement (ECGS), and heparin. To purify PAECs, a differential adhesion method was applied, wherein non-adherent cells were transferred to new gelatin-coated culture flasks 3 hours after initial seeding. Primary rat PAECs were treated with monocrotaline pyrrole (MCTP) at concentrations ranging from 10 to 50 μM for 48 to 72 hours. PAECs were identified by their characteristic cobblestone morphology and positive immunofluorescence staining for von Willebrand factor (vWF). Primary rat PAECs were treated with monocrotaline pyrrole (MCTP) at concentrations ranging from 10 to 50 μM for 48 to 72 hours. This treatment regimen aimed to induce pathological phenotypes resembling in vivo conditions, including but not limited to inflammatory activation, increased reactive oxygen species production, and endothelial dysfunction, thereby providing a reliable cellular model for investigating the molecular mechanisms of PAH. For chronic hypoxia simulation, a tri-gas incubator (controlling O₂, CO₂, and N₂) was used, typically set at 1–3% O₂ to mimic severe hypoxic conditions, with treatment durations generally lasting 24–72 hours.

**Human Embryonic Kidney 293T (HEK293T) Cells:** HEK293T cells were cultured in Dulbecco’s Modified Eagle Medium (DMEM, Gibco) supplemented with 10% FBS (Gibco) and 1% penicillin-streptomycin (Gibco). The cells were maintained at 37°C in a humidified incubator with 5% CO₂. The medium was refreshed every 2–3 days, and cells were passaged using 0.25% trypsin-EDTA upon reaching 80–90% confluence.

**Cell Treatments**
To induce autophagy, cells were subjected to nutrient deprivation by culturing in serum-free medium, low-glucose DMEM, or Earle’s Balanced Salt Solution (EBSS) for specified durations. To inhibit autophagy, cells were treated with 50 μM chloroquine (CQ) for 24 hours; other autophagy inhibitors such as 5 mM 3-methyladenine (3-MA) for 4 hours or 20 mM ammonium chloride (NH₄Cl) for 24 hours were also applied as required; For genetic manipulations, gene knockdown was achieved by transfecting cells with siRNA or shRNA oligonucleotides targeting specific genes (e.g., FSP1, NBR1, RNFT1) using Lipofectamine 3000 transfection reagent. Conversely, gene overexpression was performed by transfecting cells with plasmid vectors encoding wild-type or mutant forms of the target genes.

**Hemodynamic and Morphological Indices**

For rodent hemodynamic measurement, a Millar SPR-671 microtip pressure-volume catheter (Millar Instruments, Houston, USA) connected to PowerLab 16/35 data acquisition workstation (ADInstruments, Australia) was applied, and all pressure waveforms were recorded and analyzed via LabChart 8.0 software.

Following euthanasia, the rat heart was excised. The atria and great vessel roots were removed along the atrioventricular groove. The heart was then dissected along the posterior interventricular groove into the right ventricle (RV) and the left ventricle plus septum (LV+S). After blotting dry on filter paper, the RV free wall and the LV+S were weighed separately using an electronic balance. The right ventricular hypertrophy index (RVHI) was calculated as follows: RVHI = (mass of RV / mass of LV+S) × 100%.

Under anesthesia, a micro-catheter with a pressure sensor at its tip was inserted into a rat via the jugular or femoral vein. The catheter was gently advanced into the right ventricle under real-time pressure waveform monitoring. The right ventricular systolic pressure (RVSP) was directly and accurately recorded and calculated when the pressure waveform transitioned from the low-pressure atrial pattern to the characteristic ventricular pattern featuring a high systolic peak and low diastolic pressure. When the catheter tip was stabilized within the pulmonary artery, displaying the typical pulmonary artery pressure waveform, the mean value of the blood pressure recorded over the entire cardiac cycle represented the mean pulmonary arterial pressure (mPAP). After successful catheter placement into the pulmonary artery, the balloon at the catheter tip was inflated to temporarily occlude blood flow in the branch pulmonary artery. The pressure measured by the catheter tip at this point is the pulmonary capillary wedge pressure (PCWP), which reflects the left atrial filling pressure. Pulmonary vascular resistance (PVR) was calculated using the formula: PVR = (mPAP - PCWP) / cardiac output. This ratio reflects the resistance and stiffness of the pulmonary vascular bed.

**Assessment of Pulmonary Vascular Remodeling**
Following euthanasia, lung tissues were collected from rats and fixed with paraformaldehyde, embedded in paraffin, and sectioned. Hematoxylin and eosin (H&E) staining was first performed to observe medial thickening of small pulmonary arteries and luminal narrowing. Adjacent tissue sections were subjected to Masson's trichrome staining. The area percentage of blue collagen regions in the vascular wall was quantified using image analysis software to specifically evaluate collagen deposition within the small arteries.

**Detection of Inflammatory Status**
The protein concentrations of inflammatory cytokines, including interleukin-1β (IL-1β), tumor necrosis factor-α (TNF-α), and interleukin-6 (IL-6), in cell culture supernatants or tissue homogenates were measured using commercial enzyme-linked immunosorbent assay (ELISA) kits (R&D Systems) according to the manufacturer's instructions.

**In Vitro Tube Formation Assay**
The angiogenic capacity of PAECs was assessed using a Matrigel-based tube formation assay. Briefly, 96-well plates were coated with Growth Factor Reduced Matrigel (Corning) and allowed to polymerize at 37°C for 30 minutes. Pretreated PAECs were then seeded onto the Matrigel surface at a density of 2×10⁴ cells per well and incubated at 37°C with 5% CO₂ for 6–8 hours. Tube formation was visualized using an inverted phase-contrast microscope (Nikon). The total tube length and number of master junctions were quantified from three random fields per well using ImageJ software with the Angiogenesis Analyzer plugin.

**Analysis of eNOS/NO Signaling Pathway**
Total protein was extracted, and the expression level of endothelial nitric oxide synthase (eNOS) was detected by Western blotting. Meanwhile, the accumulation of nitrite (a stable metabolite of nitric oxide) in the culture supernatant was measured using the Griess reagent system (Promega).

**Detection of Adhesion Molecules and BMPR-II Signaling**
The protein expression levels of adhesion molecules such as ICAM-1 and VCAM-1 were assessed by Western blotting. In experiments involving the BMPR-II signaling pathway, protein lysates were immunoblotted with antibodies targeting BMPR-II (Cell Signaling Technology), and band intensities were analyzed by grayscale quantification using ImageJ software.

**Drug Safety Evaluation**
The toxicity and safety of drug treatments were evaluated by monitoring body weight, histological sections of major organs (liver, spleen, and kidney), and serum liver/kidney function indicators (ALT, AST, UREA, CREA).

**Measurement of NADP⁺ and NADPH Levels**

The levels of NADP⁺ and its reduced form, NADPH, in mouse lung tissues were measured using a commercial NADP⁺/NADPH assay kit (e.g., Beyotime Biotechnology, S0179). The concentration of NADP⁺ was calculated by subtracting the NADPH concentration from the total NADP(H) concentration. The NADP⁺/NADPH ratio was then determined for each sample.

**Assessment of Autophagic Flux**

Autophagic flux was monitored by quantifying the levels of lipidated LC3 (LC3-II) and the autophagy substrate p62 under oxidative stress conditions via Western blotting.In this experiment, autophagic activity was evaluated by analyzing the LC3-II/LC3-I ratio and the extent of p62 degradation. Alternatively, autophagic activity was examined by immunofluorescence staining: cells grown on coverslips were fixed, permeabilized, and stained with an LC3 antibody, and the formation of LC3 puncta was observed and quantified using a fluorescence microscope.

**Cell Proliferation Tracking**

Cell proliferation was tracked using the fluorescent dye Carboxyfluorescein Diacetate Succinimidyl Ester (CFDA-SE; Thermo Fisher Scientific). Briefly, cells were harvested, labeled with 5 µM CFDA-SE in PBS for 20 minutes at 37°C, and the reaction was quenched with complete medium. The dilution of CFDA-SE fluorescence, indicative of cell division, was analyzed by flow cytometry at specified time points.

**Detection of Lipid Reactive Oxygen Species (Lip-ROS)**
Intracellular lipid reactive oxygen species (Lip-ROS) were detected using the fluorescent probe C11-BODIPY 581/591 (Thermo Fisher Scientific). After treatment, cells were incubated with 2 µM C11-BODIPY 581/591 and analyzed by flow cytometry or immunofluorescence imaging. The ratio of fluorescence intensity in the oxidized state (detected in the FITC channel) to the reduced state (detected in the PE-Texas Red channel) was calculated to quantify lipid peroxidation levels.

**Detection of Ferrous Iron (Fe²⁺)**
The intracellular ferrous iron (Fe²⁺) level was measured using the FerroOrange probe (Dojindo Molecular Technologies) following the manufacturer's protocol. After incubation with 1 µM FerroOrange, the fluorescence intensity (Ex/Em = 543/580 nm) was quantified using a microplate reader or flow cytometer.

**Detection of Lipid Peroxidation**
The extent of lipid peroxidation was evaluated by quantifying the concentration of malondialdehyde (MDA), a terminal product, using a commercial Lipid Peroxidation MDA Assay Kit (Beyotime Biotechnology) based on the thiobarbituric acid (TBA) method. The MDA content was determined by measuring the absorbance at 532 nm and normalized to the total protein concentration.

**Detection of Reduced Glutathione (GSH) Level**
The intracellular level of reduced glutathione (GSH) was quantified using a GSH Assay Kit (Beyotime Biotechnology). The concentration of GSH was determined spectrophotometrically at 412 nm after reaction with DTNB and normalized to the total protein content.

**Western Blot Analysis**

Cells and brain tissue samples were lysed in ice-cold RIPA buffer containing protease inhibitor and PMSF for 30 min. Protein concentration was determined using a BCA assay kit (BB-3401, Shanghai Beibo Biotechnology). Equal amounts of protein were separated by 7.5–12.5% SDS-PAGE and transferred to PVDF membranes.

**Antibodies**

The antibodies used for western blotting were anti-FSP1 (1:1000, MA5-32347, Proteintech), anti-GAPDH (1:100000, 60004-1-Ig, Proteintech), anti-RNFT1 (1:1000, PA5-48913, Proteintech), anti-FTH1 (1:2000, ab308115, Abcam), anti-Hepcidin (1:1000, ab187778, Abcam), anti-NBR1 (1:1000, PA5-48913, Proteintech), anti-Beclin (1:1000, MA5-15825, Proteintech) , anti-BMPR2 (1:1000, MA5-15827, Proteintech), anti-LC3B(1:2000, #12513, CST)，anti-p62 (1:2000, #4858, CST), anti-ICAM-1 (1:250, MA5407, Proteintech), anti-eNOs (1:2000, PA1-037, Proteintech), anti-NCOA4 (1:5000, MA5-30728, Proteintech).

**Real time fluorescence quantitative PCR (qPCR)**

Total RNA (0.2–1 μg) was reverse-transcribed into cDNA using oligo(dT) primers. qPCR was performed using SYBR Green Premix on a 7300 Real-Time PCR System (Applied Biosystems), with amplification carried out using gene-specific primers and universal primers.

**Statistical analysis**

All data are presented as mean ± SD from ≥3 independent experiments. Correlations were analyzed using Spearman’s correlation coefficient. Survival differences were assessed by Kaplan–Meier analysis with log-rank test. Group comparisons were performed using unpaired two-tailed t-test, one-way or two-way ANOVA followed by Tukey’s post hoc test. A p-value < 0.05 was considered statistically significant. All analyses were conducted with GraphPad Prism 9.0.
